# Timeline of changes in appetite during weight loss with a ketogenic diet

**DOI:** 10.1038/ijo.2017.96

**Published:** 2017-05-16

**Authors:** S Nymo, S R Coutinho, J Jørgensen, J F Rehfeld, H Truby, B Kulseng, C Martins

**Affiliations:** 1 Faculty of Medicine, Department of Cancer Research and Molecular Medicine, Norwegian University of Science and Technology (NTNU), Obesity Research Group,, Trondheim, Norway; 2 Department of Clinical Biochemistry, Rigshospitalet, University of Copenhagen, Copenhagen, Denmark; 3 Department of Nutrition, Dietetics & Food, Monash University, Melbourne, Victoria, Australia; 4 Centre for Obesity and Innovation (ObeCe), Clinic of Surgery, St. Olav University Hospital, Trondheim, Norway

## Abstract

**Background/objective:**

Diet-induced weight loss (WL) leads to increased hunger and reduced fullness feelings, increased ghrelin and reduced satiety peptides concentration (glucagon-like peptide-1 (GLP-1), cholecystokinin (CCK) and peptide YY (PYY)). Ketogenic diets seem to minimise or supress some of these responses. The aim of this study was to determine the timeline over which changes in appetite occur during progressive WL with a ketogenic very-low-energy diet (VLED).

**Subjects/methods:**

Thirty-one sedentary adults (18 men), with obesity (body mass index: 37±4.5 kg m^−2^) underwent 8 weeks (wks) of a VLED followed by 4 wks of weight maintenance. Body weight and composition, subjective feelings of appetite and appetite-related hormones (insulin, active ghrelin (AG), active GLP-1, total PYY and CCK) were measured in fasting and postprandially, at baseline, on day 3 of the diet, 5 and 10% WL, and at wks 9 and 13. Data are shown as mean±s.d.

**Results:**

A significant increase in fasting hunger was observed by day 3 (2±1% WL), (*P*<0.01), 5% WL (12±8 days) (*P*<0.05) and wk 13 (17±2% WL) (*P*<0.05). Increased desire to eat was observed by day 3 (*P*<0.01) and 5% WL (*P*<0.05). Postprandial prospective food consumption was significantly reduced at wk 9 (16±2% WL) (*P*<0.01). Basal total PYY was significantly reduced at 10% WL (32±8 days) (*P*<0.05). Postprandial active GLP-1 was increased at 5% WL (*P*<0.01) and CCK reduced at 5 and 10% WL (*P*<0.01, for both) and wk 9 (*P*<0.001). Basal and postprandial AG were significantly increased at wk 13 (*P*<0.001, both).

**Conclusions:**

WL with a ketogenic VLED transiently increases the drive to eat up to 3 weeks (5% WL). After that, and while participants are ketotic, a 10–17% WL is not associated with increased appetite. However, hunger feelings and AG concentrations increase significantly from baseline, once refeeding occurs.

## Introduction

Obesity has reached epidemic proportions worldwide and is associated with negative public health consequences and large socioeconomic costs.^1^ A weight loss (WL) between 5 and 10% of baseline weight, if sustained, can have large health benefits by preventing or improving several obesity-related risk factors and comorbidities.^2^ Even though this can be achieved in the short-term with lifestyle interventions,^3^ most adults will experience weight regain in the long term.^3, 4, 5^

Maintaining lost weight is physiologically challenging, as diet-induced WL is associated with compensatory responses on both sides of the energy balance equation.^6^ These responses are driven by a cross talk between the gut and the brain, leading to an increase in appetite and reduction in satiety,^7, 8, 9^ concomitant with an overall reduction in energy expenditure.^10, 11, 12^ These mechanisms can reduce WL rate and increases the risk of relapse.^9^ An increase in ghrelin plasma concentration^13, 14, 15^ and a reduction in the concentration of several satiety hormones, such as peptide YY (PYY), glucagon-like peptide-1 (GLP-1)^13, 16, 17, 18^ and cholecystokinin (CCK)^13, 19^ has also been reported with diet-induced WL.

To our knowledge, no studies to date have determined how appetite is affected with progressive WL. With a WL of only 1–2 kg being shown to increase subjective feelings of hunger,^20^ and a WL of 0.8 kg associated with an increase in postprandial total ghrelin and a reduction in subjective feelings of fullness,^21^ it is not surprising that adults find WL maintenance extremely hard. However, when carbohydrates and/or energy (using a very-low-energy diet (VLED)) are restricted sufficiently to induce ketosis, the increase in appetite seen with WL appears to be absent.^12, 22^ This has contributed to the generalised idea that ketogenic diets are easy to follow. Most of the studies looking at the impact of WL induced with ketogenic diets on appetite have, nevertheless, been done in females^22^ and none has investigated how long time does it take for appetite suppression to occur under ketogenic diets. Therefore, the primary aim of this study was to assess the timeline over which changes in both subjective feelings of appetite and appetite-related hormones are activated during WL with a ketogenic VLED. A secondary aim was to assess if males and females respond differently.

## Materials and methods

### Participants

Healthy adults with obesity (30<BMI <45 kg m^−2^) were recruited via newspaper advertising serving the community of Trondheim, Norway. The study was approved by the regional ethics committee (Ref., 2012/1901). The study was registered in ClinicalTrial.gov (NCT01834859), and conducted according to the guidelines laid down in the Declaration of Helsinki. All participants provided written informed consent before commencement.

Participants were required to be weight stable (<2 kg body weight change over the last 3 months), not currently dieting to lose weight, and with a sedentary lifestyle (not engaged in strenuous work or in regular brisk leisure time exercise more than once a week or in light exercise for more than 20 min per day in more than 3 times per week). Due to the known effect of phase of menstrual cycle on appetite^23^ females had to be postmenopausal or taking hormonal contraceptives. Exclusion criteria were pregnancy, breastfeeding and clinically significant illness including diabetes, previous WL surgery and/or medication known to affect appetite/metabolism or induce WL.

### Study design

This was a longitudinal intervention study with repeated measurements. Participants were provided with an 8-week ketogenic VLED, followed by 4 weeks of weight stabilisation, and were requested not to change their physical activity levels throughout the study (Supplementary Figure I).

### Detailed protocol

#### Weight loss phase

Participants followed for 8 weeks a ketogenic VLED (Allevo, Karo Pharma AS, Sweden) with 550/660 kcal per day for females and males, respectively (macronutrient composition: carbohydrates 42%, protein 36%, fat 18% and fibre 4%). No energy fluids were allowed *ad libitum*. Intake of low-starch vegetables (max 100 g per day) was encouraged, to provide dietary fibre.

### Weight stabilisation phase

At week 9, participants were gradually introduced to normal food, while reducing intake of the VLED products. An individual diet plan was prescribed by a trained dietician tailored to individual energy requirements (measured resting metabolic rate (RMR) × physical activity level (PAL) (extracted from individual physical activity monitors (BodyMedia, SenseWear, Pittsburgh, PA, USA) at week 8)), with 15–20% protein, 20–30% fat and 50–60% carbohydrates, aimed at weight stabilisation.^24^

### Objective measures of compliance

*Diet*: Participants met every week for an individual 20 min consultation with a dietician, to review their food records. Urine acetoacetic acid concentration was also measured weekly, using Ketostix reagent strips. Participants who were not ketotic on more than one occasion were considered not compliant and were excluded from the analysis. Concentration of plasma β-hydroxybutyric acid (β-HB) in the fasting state was also measured with a Ketone Body Assay Kit (Mark134, Sigma-Aldrich, St Louis, MO, USA) at baseline, day 3, 5 and 10% WL and weeks 9 and 13.

*Physical activity*: Armbands were used for 7 days at baseline, weeks 4, 8 and 12. Data were considered valid if participants wore the device for ⩾4days, including at least 1 weekend day, on more than 95% (22.8 h per day) of the time.^25^

### Data collection

The following measurements were performed in fasting at baseline, day 3 of the VLED, when each individual participant reached 5 and 10% WL, and at weeks 9 (the day immediately after the end of the VLED) and 13.

#### Body weight and composition

Air displacement plethysmography (BodPod, COSMED, Rome, Italy) was used.

#### Appetite measures

Subjective appetite feelings (hunger, fullness, desire to eat and prospective food consumption (PFC)) were measured using a validated 10 cm visual analogue scale^26^ and blood samples were collected in fasting and every 30 min (0, 30, 60, 90, 120 and 150 min) after a standardised breakfast (600 kcal: 17% protein, 35% fat and 48% carbohydrates), for a period of 2.5 h. Plasma samples were analysed for active ghrelin (AG), total PYY, active GLP-1 and insulin using a Human Metabolic Hormone Magnetic Bead Panel (LINCOplex Kit, Millipore, St Louis, MO, USA) and CCK using an ‘in-house’’ RIA method^27^ (intra- and inter-assay coefficient of variation were <10% and <20% for AG, GLP-1 and PYY; <10% and <15% for insulin and <5% and <15% for CCK, respectively).

### Power calculation

Sample size estimation was based on expected changes (from baseline) in fasting AG plasma concentration overtime (day 3: 27; 5 and 10%: 0; week 9: 4 and week 13: 53 pmol l^−1^). Having into consideration that no previous studies have been done in this area, we used data from Sumithran *et al.*^12^ for weeks 9 and 13 and hypothesised that there would be an increase in AG on day 3 (approximately half of that seen on week 13) and no changes at 5 and 10% WL (given that participants would be under ketosis). For a SD of 89 pmol l^−1^,^12^ a power of 80%, a significance level of 5% and assuming a low correlation between time points (*r*=0.3), 32 participants would be necessary.

### Statistical analysis

Statistical analysis was performed with SPSS version 22 (SPSS Inc., Chicago, IL, USA), and data presented as estimated marginal means±s.e.m., with the exception of BMI, age, average time to achieve 5 and 10% WL and WL (%) at day 3, weeks 9 and 13, where means±s.d. are given. Statistical significance was set at *P*<0.05. Data were analysed using linear mixed-effects models, with restricted maximum likelihood estimation, including fixed effects for time and sex, and their interaction. Bonferroni correction was used for *post hoc* pairwise comparisons. The Benjamini—Hochberg method, which controls for the false discovery rate was used to adjust for the fact that we have looked at a large number of outcome variables.^28^ Analyses of fasting and 2.5-h postprandial hormone profile for AG and subjective feelings of hunger were also carried out by linear mixed-effects models (fixed effects for sampling time points (0, 30, 60, 90, 120 and 150 min), time (baseline, day 3, 5 and 10% WL and wks 9 and 13), sex and interactions).

Weight at baseline was used as a covariate in the linear mixed-effects models when looking at changes in subjective and objective measures of appetite. Given that this did not change the significance of the results; the unadjusted values are presented. Total area under the curve for subjective feelings of appetite and appetite hormones was calculated from 0 to 150 min using the trapezoid rule. Participants with available data on at least three out of the six time points were considered completers.

## Results

### Participants

Thirty-three participants met study entry criteria and 31 (13 females, 6 postmenopausal) were included in the analysis (one female withdrew due to personal reasons and one male due to not tolerating the VLED (verified incidence of vomiting, dizziness and fatigue)). Completers had an average BMI of 36.7±4.5 kg m^−2^ and a mean age of 43±10 years. Males were heavier and with greater FFM (kg) than females (*P*<0.01 and *P*<0.001, respectively), but there were no significant differences in age or BMI between sexes (Supplementary Table I).

### Objective measures of compliance

**Figure 1:**
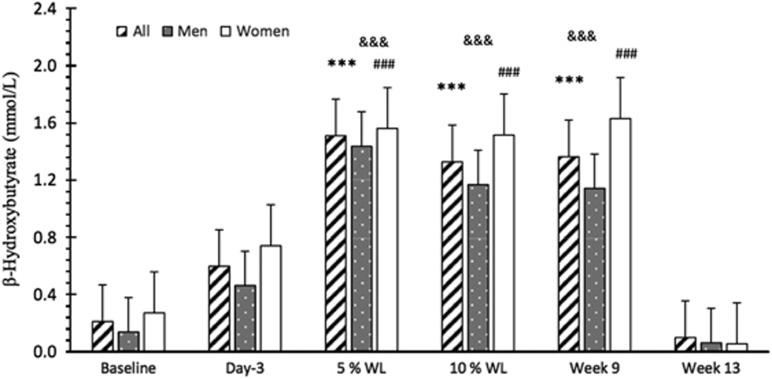
Basal plasma concentration of β-hydroxybutyric acid over time in all participants, males and females. Results presented as estimated marginal means±s.e.m. A significant main effect of time was found for β-hydroxybutyrate (*P*<0.001). Symbols denote significant differences from baseline in all participants ***P<0.001, males ^&&&^*P*<0.001 and females ^###^*P*<0.001. WL, weight loss

*Diet*: Compliance with the VLED was excellent and no participant was excluded based on not being compliant. Participants were already ketotic at day 3 (0.60±0.13mmol l^−1^ of β-HB), even though β-HB plasma concentrations were only significantly increased, compared with baseline, at 5% WL (12±8 days) (*P*<0.001 for all, males and females), and continued increased up to week 9 (16±2% WL) (*P*<0.001 for all, males and females) (Figure 1).

*Physical activity*: All participants were sedentary at baseline and there were no significant changes over time in any PA variable studied (Supplementary Table II).

### Body weight and composition

**Figure 2:**
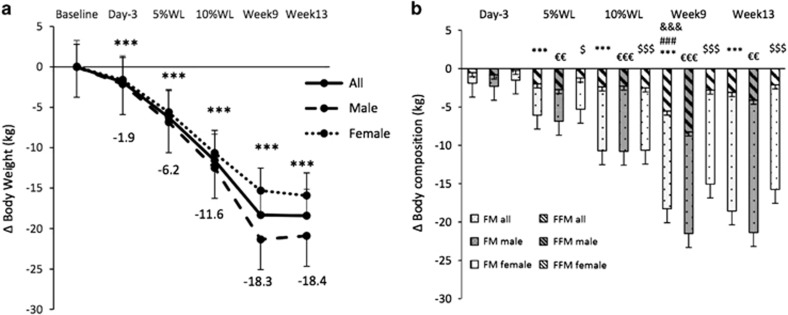
Changes in body weight (**a**) and body composition (**b**) over time in all participants, males and females. Results are presented as estimated marginal means±s.e.m. Symbols denote significant changes from baseline (all participants: ***P<0.001 for body weight and FM and ^###^*P*<0.001 for FFM; males: ^&&&^*P*<0.001 for FFM and ^∈∈∈^*P*<0.001, and ^∈∈^*P*<0.01 for FM and females: ^$$$^*P*<0.001 and ^$^*P*<0.05 for FM). FFM, fat-free mass; FM, fat mass; WL, weight loss.

A significant main effect of time, sex and interaction (*P*<0.001, *P*<0.05 and *P*<0.001, respectively) was found for body weight. WL was already significant (*P*<0.001) on day 3 in all participants and males (2±1% WL for both) and body weight continued decreasing progressively, but with no significant differences between week 9 and 13 (16±2% and 17±2% WL, respectively) (Figure 2a). About 5% WL was achieved on day 12±6 (11±5 and 15±7, for males and females, respectively) and 10% WL on day 32±8 (28±7 and 37±6 for males and females, respectively). Overall WL (kg) was significantly larger in males compared with females (*P*<0.001), and also at weeks 9 and 13 (*P*<0.001 for both). When WL was expressed in % there were no overall significant sex differences (*P*=0.053), but at week 9 (*P*<0.01) males had a significantly larger WL than females.

FM (kg) decreased significantly for the first time after 5% WL (12±8 days) (*P*<0.001 for all participants, *P*<0.01 for males and *P*<0.05 for females) and was significantly lower than baseline at all the other subsequent time points (Figure 2b).

There was a significant decrease in FFM (kg) for the first time at week 9 (16±2% WL), in all participants and in males (5.8±1.0 kg and 8.3±1.4 kg, respectively, *P*<0.001 for both). FFM (kg) was never significantly lower than baseline in females (Figure 2b).

### Appetite feelings

**Figure 3:**
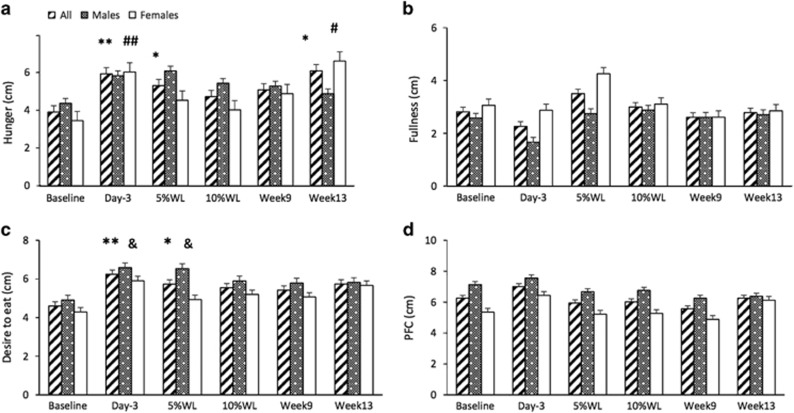
Subjective feelings of appetite (**a**: hunger, **b**: fullness, **c**: desire to eat and **d**: PFC) in fasting, over time, in all participants, males and females. Results presented as estimated marginal means±s.e.m. Symbols denote significant differences from baseline in all participants: **P<0.01 and *P<0.05, males: ^&^*P*<0.05 and females: ^##^*P*<0.01 and ^#^*P*<0.05. PFC, prospective food consumption; WL, weight loss.

Fasting feelings of appetite in all participants and by sex are reported in Figure 3.

A significant main effect of time, but no main effect of sex or interaction, was found for hunger and desire to eat in fasting (*P*<0.01, for both). Feelings of hunger in fasting were significantly increased, compared to baseline, at day 3 (2±1% WL) (*P*<0.01), after 5% WL (12±8 days) (*P*<0.05) and at week 13 (17±2% WL) (*P*<0.05) in all participants. In women, fasting hunger was significantly increased on day 3 and week 13 (*P*<0.01 and *P*<0.05, respectively). In men, no significant changes were seen at any time point. Feelings of desire to eat were significantly increased at day 3 and after 5% WL in all participants (*P*<0.01 and *P*<0.05, respectively) and males (*P*<0.05 for both). For feelings of PFC in fasting, there was a significant main effect of time only (*P*<0.05). However, no significant differences were found between baseline and any other time point. Men reported greater feelings of PFC in fasting overall (6.8 ±0.4 vs 5.5±0.4 cm, *P*=0.05). No significant main effect of time, sex or interaction was found for feelings of fullness in fasting.

**Figure 4:**
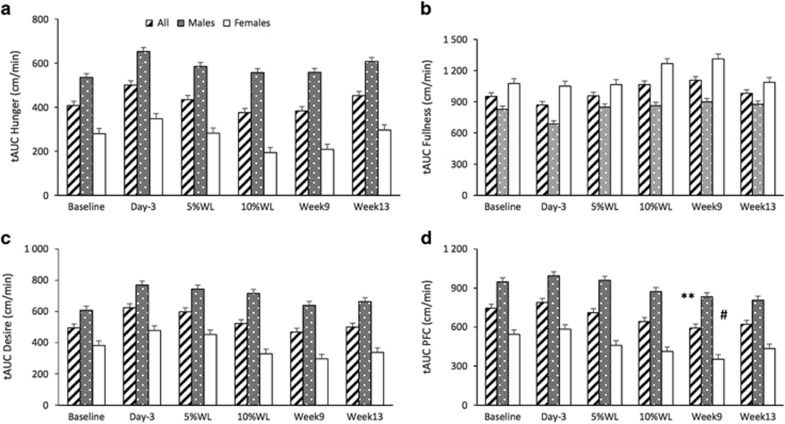
AUC for subjective feelings of appetite (**a**: hunger, **b**: fullness, **c**: desire to eat and **d**: PFC) over time in all participants, males and females. Results presented as estimated marginal means±s.e.m. Symbols denote significant differences from baseline in all participants: **P<0.01 and females: ^#^*P*<0.05. AUC, total area under the curve; PFC, prospective food consumption; WL, weight loss.

AUC for subjective feelings of appetite overtime is reported in Figure 4. A significant main effect of sex, but no main effect of time or interaction was found for hunger AUC (*P*<0.01). A significant main effect of time and sex was found for fullness AUC (*P*<0.01 and *P*<0.001, respectively), desire to eat (*P*<0.05 for both) and PFC (*P*<0.001 and *P*<0.01, respectively). No significant differences were found between baseline and any other time point for hunger AUC, desire to eat or fullness. PFC AUC was significantly lower at week 9 (16±2% WL), compared with baseline in all participants and females (*P*<0.01 and *P*<0.05, respectively). Males had significantly higher overall hunger AUC (582.2±52.4 vs 267.18±61.8 cm min^−1^, respectively), PFC AUC (904.1±80.9 vs 459.8±95.2 cm min^−1^, respectively) and desire to eat (689.1±72.1 vs 380.1± 84.8 cm min^−1^, respectively), compared to females. Females had significantly higher fullness AUC (832.6±35.9 vs 1149.7± 42.3 cm min^−1^, respectively), compared to males.

Fasting and 2.5-h postprandial hunger feelings can be seen in Supplementary Figure II.

### Appetite regulating hormones

**Figure 5:**
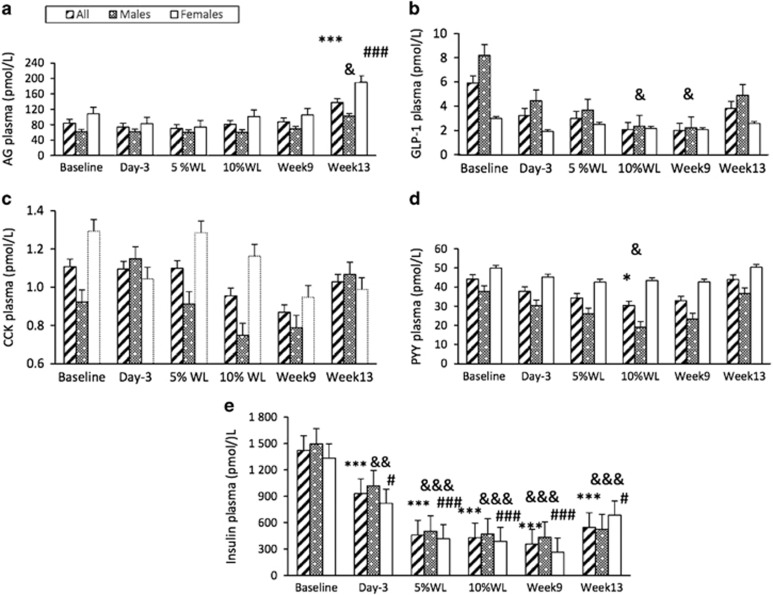
Basal plasma concentrations of appetite-related hormones (**a**: active ghrelin (AG), **b**: GLP-1, **c**: CCK, **d**: PYY and **e**: insulin) over time in all participants, males and females. Results presented as estimated marginal means±s.e.m. Symbols denote significant differences from baseline in all participants: ***P<0.001 and *P<0.05, males: ^&&&^*P*<0.001, ^&&^*P*<0.01 and ^&^*P*<0.05, and females: ^###^*P*<0.001 and ^#^*P*<0.05. CCK, cholecystokinin; GLP-1, glucagon-like peptide-1; PYY, total peptide YY; WL, weight loss.

Basal plasma concentration of appetite-related hormones overtime is reported in Figure 5. There was a significant main effect of time, sex and interaction (*P*<0.001, *P*<0.01, *P*<0.05, respectively) for basal AG. There was a significant increase in basal AG compared to baseline only on week 13 (16±2% WL) in all participants, females (*P*<0.001 for both) and males (*P*<0.05). Basal AG concentration was significantly lower overall in males compared with females (69.2±8.3 vs 110.3±10.2 pmol l^−1^, respectively). No significant main effects of time, sex or interaction were found for basal active GLP-1 or CCK. A significant main effect of time (*P*<0.05), but no main effect of sex or interaction was found for basal total PYY. A significant decrease in basal active GLP-1 was seen at 10% WL (32±8 days) and at week 9 (16% WL), compared with baseline, in males only (*P*<0.05 for both). A significant reduction in basal PYY was present only after 10% WL (32±8 days), when compared with baseline, in all participants and males (*P*<0.05 for both). A significant main effect of time (*P*<0.001), but no effect of sex or interaction was found for basal insulin. There was a significant reduction in basal insulin, compared to baseline, for the first time at day 3, in all participants, males and females (*P*<0.001, *P*<0.01 and *P*<0.05, respectively), which persisted thereafter.

**Figure 6:**
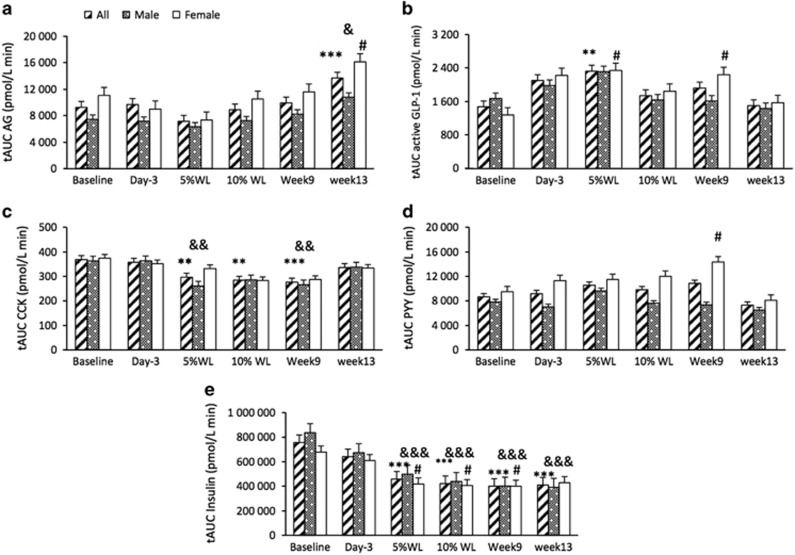
AUC for appetite-related hormones (**a**: active ghrelin (AG), **b**: GLP-1, **c**: CCK, **d**: PYY and **e**: insulin) over time in all participants, males and females. Results presented as estimated marginal means±s.e.m. Symbols denote significant differences from baseline in all participants: ***P<0.001, **P<0.01, males: ^&&&^*P*<0.001, ^&&^*P*<0.01 and ^&^*P*<0.05 and females: ^#^*P*<0.05. CCK, cholecystokinin; GLP-1, glucagon-like peptide-1; PYY, total peptide YY; tAUC, total area under the curve; WL, weight loss.

AUC for appetite-related hormones overtime is reported in Figure 6. A significant main effect of time was found for AG AUC (*P*<0.001). AG AUC was significantly increased at week 13 (17±2% WL) in all participants, males and females (*P*<0.001 for all participants and *P*<0.05 for males and females). A significant main effect of time, but no sex or interaction, was seen for active GLP-1, insulin and CCK AUC (*P*<0.01, *P*<0.001 and *P*<0.001, respectively). Active GLP-1 AUC was only significantly increased after 5% WL (12±8 days) in all participants (*P*<0.01). In females, GLP-1 AUC was significantly higher than baseline at 5% and week 9 (16±2% WL) (*P*<0.05, for both). CCK AUC was significantly reduced at 5 and 10% WL (32±8 days) (*P*<0.01 for both), and week 9 (*P*<0.001) in all participants and 5% WL and week 9 in males (*P*<0.01 for both). A significant main effect of time (*P*<0.05), but no main effect of sex or interaction, was found for PYY AUC. There was only a significant increase between baseline and week 9 (*P*<0.05) in females. Insulin AUC was significantly reduced at all time points except day 3 in all participants and males, (*P*<0.001 for all) and in females at 5 and 10% WL and week 9 (*P*<0.05 for all).

Fasting and 2.5-h postprandial AG concentrations can be seen in Supplementary Figure III.

## Discussion

In this novel study investigating progressive changes in appetite in adults during a ketogenic WL diet, both subjective feelings of hunger and desire to eat were significantly increased at day 3 (2±1% WL), but were not accompanied by significant changes in physiological appetite hormones except for insulin. This occurred despite participants being already ketotic (β-HB plasma concentration 0.60±0.13 mmol l^−1^). Previous studies looking at the impact of an acute period of energy restriction on appetite in people with normal weight and overweight (ranging from 1 to 4 days; between 60 and 85% energy restriction and inducing a WL between 0.8 and 2.4 kg) report an increase in feelings of hunger (desire to eat and PFC), both in the fasting and postprandial state.^29, 30, 31, 32^ Interestingly, these were not accompanied by changes in either AG,^29^ total ghrelin^31, 32^ or GLP-1,^29^ which is in line with our findings. However, the study from Pasiakos *et al.*^21^ showed a significant increase in postprandial total ghrelin after 2 days on a 321 kcal diet in individuals with normal weight (12 males and 1 female). The different hormone fraction, lower BMI, lower energy intake and different sex distribution compared to our study probably accounts for some of these differences. However, in our study sex responses were apparent. Males (which would be more easily comparable with Pasiakos and colleagues study^33^), displayed no changes in AG.^21^ Unfortunately, none of the previously mentioned studies measured ketosis during WL.

After 5% WL (12±8 days), feelings of hunger and desire to eat in fasting remained elevated, GLP-1 AUC had increased significantly and CCK AUC was significantly reduced. After 10% WL (32±8 days), no significant changes in subjective feelings of appetite were reported, while basal GLP-1 (in males only), basal PYY (in all and males) and CCK AUC (in all) were significantly reduced. Previous studies using ketogenic diets and inducing a similar WL (6–8%) have systematically shown no change in hunger feelings.^18, 34, 35, 36, 37, 38, 39^ Even though some of these studies report a significant increase in fullness feelings (both fasting and postprandial),^36, 38^ others report no changes in feelings of fullness in the fasting state after WL.^34, 35, 37^

A study by Adams *et al.*,^16^ where participants with obesity (males and females) underwent 6 weeks of a VLED (7% WL), reported a reduction in hunger at 90 and 120 min, an increase in fullness at 120 min postprandial, as well as a decrease in postprandial total GLP-1 (but no changes in fasting). Diepvens *et al.*^39^, where females with overweight experienced an 11% WL with a VLED, reported a significant reduction in basal concentration of AG and no changes in CCK or GLP-1 concentration (either fasting or postprandial). Similar to us, Soenen *et al.*^18^ reported a significant reduction in basal concentration of PYY after a 6% WL (4 weeks VLED).

A recent meta-analysis by Gibson *et al.* reported that losing weight with a ketogenic diet is associated with a reduction in hunger and increase in fullness feelings. All but one of the studies included in the analyses had average β-HB plasma concentration around 0.50 mmol l^−1^.^22^ It is surprising that hunger feelings in fasting in our study were increased on day 3 and 5% WL, but not at 10% WL, even though β-HB concentration did not differ from 5% WL. The reasons for this mismatch between β-HB concentration and subjective appetite feelings are unknown. However, it can be that participants got used to feeling hungry and that this perception therefore was attenuated overtime. Moreover, a recent review has suggested that other factors such as free fatty acids, reactive oxygen species and microbiota may also play a role.^40^

At week 9, with a 16±2% WL and ketosis, no changes in subjective feelings of appetite were seen, with the exception of PFC AUC, which was reduced compared to baseline in all participants and females. Basal active GLP-1 was significantly reduced in males and GLP-1 AUC was increased in females, while CCK AUC was reduced in all participants and males. Only a few studies have looked at the impact of a WL>10% under ketosis on appetite.^12^ In the systematic review and meta-analysis from Gibson *et al.*^22^, a significant increase in fullness, and decrease in hunger feelings were reported while ketotic, with a WL ranging from 5 to 12.5 kg. After 4 weeks of weight stabilisation and without ketosis, the only significant changes observed were increased hunger feelings in fasting (in all participants and females) and increased basal and postprandial concentration of AG.

Sumithran *et al.*^12^, in a similar WL intervention, reported no changes in subjective feelings of appetite and a significant decrease in basal active GLP-1, but different from our study a significant decrease in basal PYY and CCK, and a significant decrease in postprandial concentration of PYY at week 9 under ketosis. After 2 weeks of weight stabilisation and no ketosis, appetite feelings were increased. Both basal and postprandial concentration of AG were also increased, which is in line with our findings. Opposite to our findings, both basal active GLP-1 and CCK plasma concentration, and postprandial PYY and CCK concentration were reduced.^10^ This discrepancy may be due to differences in the duration of the weight stabilisation period and/or lack of power. It is possible that more than 2 weeks of weight stabilisation are needed for the concentration of satiety hormones to normalise. Another study by Chearskul *et al.*^19^ with an 8-week VLED (429 kcal per day) in 12 males with obesity who experienced a 15% WL, did not report any changes in hunger or satiety feelings, or CCK concentration, either fasting or postprandial, immediately after the intervention, while in ketosis. However, 1 week later and without ketosis, a significant reduction in postprandial CCK was described (opposite to our study where no changes were seen). Again, differences in the duration of the stabilisation phase might have impacted on the results. In a recently published study, Iepsen *et al.*^41^ reported a significant increase in fasting and postprandial total ghrelin, and PYY_3–36_ and postprandial concentration of total GLP-1 after a 17% WL induced with an 810 kcal per day diet over 8 weeks when participants were out of ketosis. Differences in the hormones’ fractions measured may account for some of the differences.

The increased hunger feelings and AG plasma concentrations seen after refeeding (week 13) in the present study may have important implications regarding WL outcomes. A recent study has reported that the increase in appetite seen with WL was three times larger than the corresponding reduction in total energy expenditure, highlighting the important role the feedback control of energy intake may have on long-term maintenance of a reduced body weight.^33^

Throughout the study period, a mismatch was apparent between subjective appetite feelings and anticipated relevant appetite-related hormones. Among others, there was an increase in hunger on day 3 (2±1% WL), despite no changes in appetite hormones except for insulin, and 5% WL, despite an increase in GLP-1 AUC and a reduction in CCK. Even though this mismatch can derive from lack of power, it is well known that the appetite control system is extremely complex and subjective feelings of appetite are not always correlated with the concentration of appetite-related hormones.^42^ Pre-lunch plasma concentrations of total ghrelin and PYY were also found not to be associated with lunch energy intake in healthy women.^43^ Moreover, a recent review by Holt *et al.*^44^ concluded that self-reported appetite ratings of appetite cannot reliably predict subsequent energy intake.

As previously described, some sex differences were noted in both subjective feelings of appetite and appetite hormones with progressive WL. The fact that males, as opposed to females, reported no increase in hunger throughout the study (despite increased AG at week 13 (16±2% WL)) is surprising. Females were found to have a more sensitive neuronal response to food-related visual cues, which can contribute to the sex differences reported in the present study. Moreover, in our study, females had overall higher fullness AUC, and overall higher basal and AUC for AG, while males had overall higher hunger, desire to eat and PFC AUC. This is in line with previous findings showing that females have a higher satiating response to meals^45^ and lower ratings of hunger and PFC.^46^ It needs to be acknowledged, nevertheless, that with the VLED used in this study, males had a much larger energy deficit per day compared with females, which resulted in a larger overall absolute WL, and this might have contributed to the sex differences reported.

A strength of this study is its design; the fact that multiple measurements were undertaken during progressive WL under ketosis, and after a period of weight stabilisation and no ketosis. This is important, given that ketosis is thought to modulate appetite.^12, 22^ Moreover, subjective and objective aspects of appetite were assessed, both in fasting and after a meal. Compliance with the intervention was monitored throughout and was excellent. The choice of time points is also a strength, given that it allowed to evaluate the impact of minimal, but significant WL (achieved at day 3), 5 and 10% WL, considered necessary to achieved health benefits,^2^ and large WL (16–17%) under ketosis vs no ketosis, on appetite outcomes. It is also a strength that we have adjusted for the increased risk of finding statistically significant differences by chance alone (by using Bonferroni adjustment for multiple time comparisons and the Benjamini—Hochberg method for multiple outcome variables).^28^ A limitation of this study is that it may be underpowered to examine sex differences.

This study has important practical implications for patients, clinicians and researchers alike. Opposite to previous reports that the drive to eat is suppressed while under a ketogenic diet,^19, 22^ we have shown a transitory increase in hunger from baseline up to 3 weeks on a ketogenic VLED, which then disappear and only come back after refeeding (and no ketosis). This information is of importance because it can influence patients’ expectations and adherence to VLEDs.

## Conclusions

Progressive WL with a ketogenic VLED induces a transient increase in fasting hunger feelings up to 5% WL (3 weeks), despite no changes in AG and an increase in active GLP-1 AUC. A WL between 10 and 17% under ketosis is not associated with increased appetite feelings or AG plasma concentration, despite reduced fasting concentration of total PYY and CCK AUC. Increased hunger and AG plasma concentration return with refeeding (no ketosis) and weight stabilisation. Sex seems to modulate some of the changes in appetite seen with WL, however, larger studies powered to detect sex differences are required to confirm these findings.
