# Supplementary material for: Timeline of changes in appetite during weight loss with a ketogenic diet
Source: Int J Obes (Lond). 2017 May 16;41(8):1224–31. doi: 10.1038/ijo.2017.96 (PMC5550564; doi:10.1038/ijo.2017.96)
Supplement: Supplementary Information [file ijo201796x1.docx]

**Supplementary figure I.** Study-diagram**.** Data collection was done see arrows. VLED: very low energy diet, ADP: Air-displacement plethysmography. β-HB : β-hydroxybutyric acid. ^*^ See solid arrows; ^#^ See dashed arrows.

**Supplementary figure II:** Mean fasting and postprandial ratings of hunger feelings in all participants (A), males (B) and females (C) at baseline, day 3, 5 and 10 % WL, weeks 9 and 13. WL: weight loss. LMM revealed a significant main effect of sampling time, assessment time and sex (P<0.001 for all), but no significant interactions. Postprandial hunger feelings were significantly reduced 15 min after breakfast, with no further significant reductions (and hunger was lower at all time points compared with fasting levels, P<0.001 for all) Mean hunger values were significantly increased at day 3 (P<0.001) and 5 % WL (P<0.01) only. Males had an overall hunger feeling compared with females (3.73±0.29 cm vs. 1.97±0.34 cm, respectively).

**Supplementary figure III:** Mean fasting and postprandial plasma concentrations of AG for all participants (A), males (B) and females (C) at baseline, day 3, 5 and 10 % WL, weeks 9 and 13. AG: active ghrelin. WL: weight loss. LMM revealed a significant main effect of sampling time, assessment time (P<0.001 for both), sex (P<0.05) and an interaction between sex and assessment time (P<0.001). Postprandial AG concentrations were significantly reduced at 30 min postprandially, with a further reduction at 60 min (P<0.001, for both) and no further reductions afterwards (AG was lower at all-time points compared to basal concentration (P<0.001 for all)). AG concentrations were significantly increased at week 13 compared with baseline at 30 min and 90 min (P<0.01 for both), at 120 min between baseline and 5 % WL and week 13 (P<0.05 and P<0.01, respectively) and 150 min between baseline and 5 % WL (P<0.05).

Mean AG concentrations were significantly reduced compared with baseline at day 3 (P<0.01), 5 % WL (P<0.01), and significantly increased at week 13 (P<0.001) for all participants and females, but only an increase at week 13 in males (P<0.001).

Mean AG concentrations were significantly higher in females compared to males (76.4±8.0 pmol/L vs.54.8±6.5 pmol/L, respectively).
