# Supplementary figures and images for: Timeline of changes in appetite during weight loss with a ketogenic diet
Source: Int J Obes (Lond). 2017 May 16;41(8):1224–31. doi: 10.1038/ijo.2017.96 (PMC5550564; doi:10.1038/ijo.2017.96)

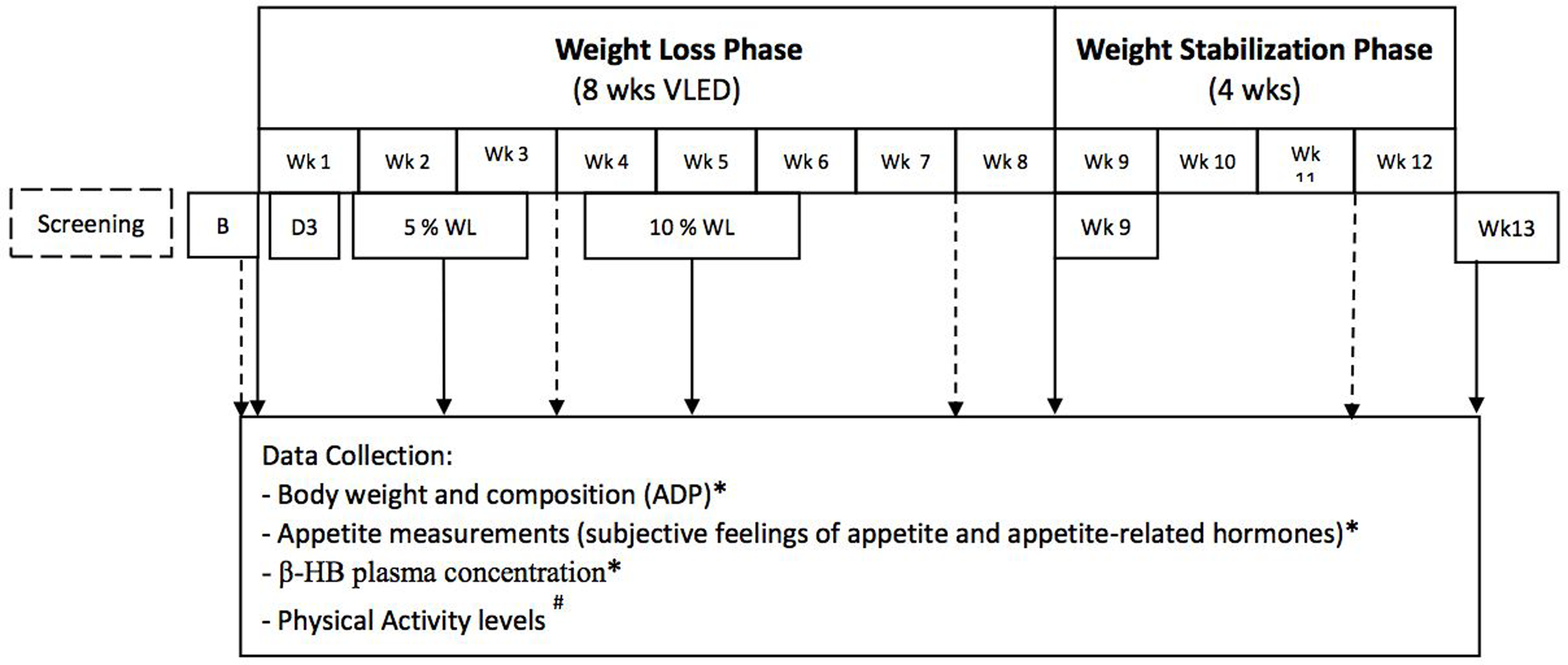

Supplement: Supplementary Figure 1 [file ijo201796x2.tif]

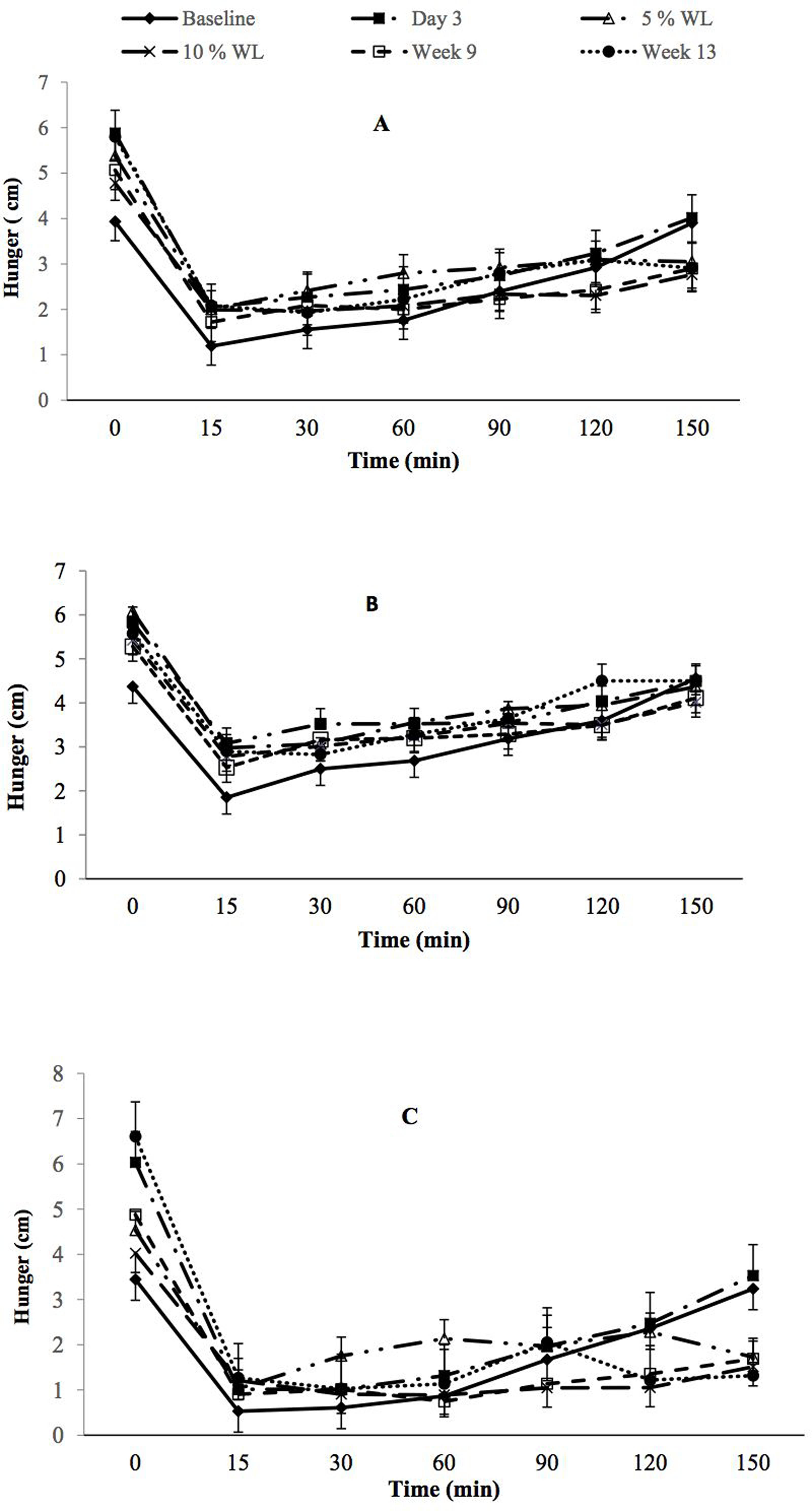

Supplement: Supplementary Figure 2 [file ijo201796x3.tif]

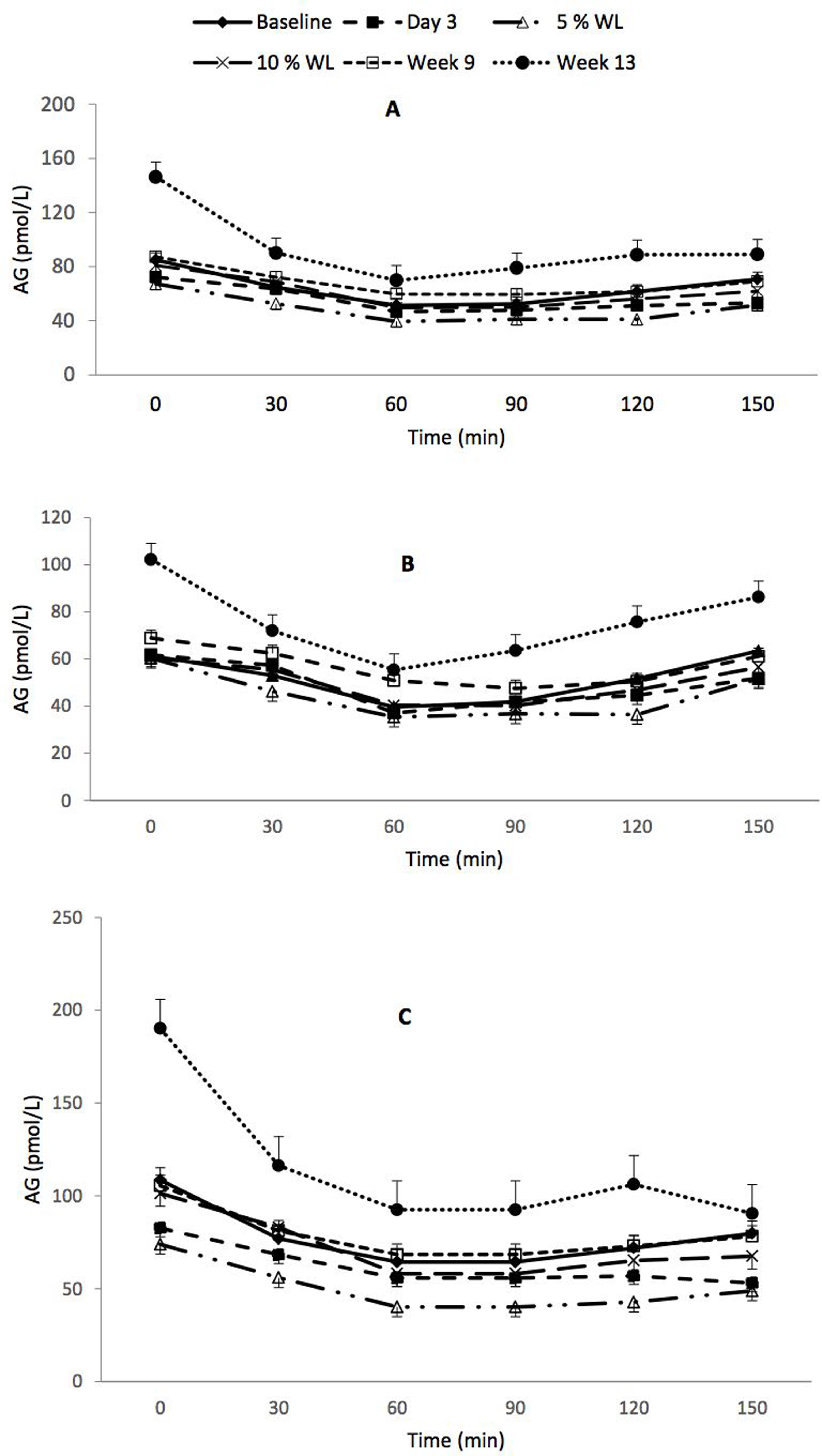

Supplement: Supplementary Figure 3 [file ijo201796x4.tif]
