# Supplementary material for: Timeline of changes in appetite during weight loss with a ketogenic diet
Source: Int J Obes (Lond). 2017 May 16;41(8):1224–31. doi: 10.1038/ijo.2017.96 (PMC5550564; doi:10.1038/ijo.2017.96)
Supplement: Supplementary Table 1 [file ijo201796x5.docx]

| **Supplementary table I.** **Baseline characteristics of all participants, males and females** | | | |
| --- | --- | --- | --- |
|  | **All** | ♂ | ♀ |
|  | **(N=31)** | **(n=18)** | **(n=13)** |
| Age (years) | 43.1±10.2 | 40.5±7.9 | 46.8±12.1 |
| Weight (kg) ^*^ | 114.8±20.0 | 123.4±18.0 | 102.7±16.3 |
| BMI (kg/m^2^) | 36.7±4.5 | 36.8±4.8 | 36.5±4.4 |
| FM (kg) | 47.8±10.8 | 46.2.5±10.9 | 50.0±10.7 |
| FFM (kg) ^**^ | 65.3±13.5 | 75.1±7.9 | 52.5±6.3 |
| Results are expressed as mean ± SD. BMI: Body Mass Index. FM: Fat Mass, FFM: Fat Free Mass. Symbols denote significant gender differences: **P<0.001, *P<0.01 | | | |
