# Supplementary material for: Timeline of changes in appetite during weight loss with a ketogenic diet
Source: Int J Obes (Lond). 2017 May 16;41(8):1224–31. doi: 10.1038/ijo.2017.96 (PMC5550564; doi:10.1038/ijo.2017.96)
Supplement: Supplementary Table 2 [file ijo201796x6.docx]

| **Supplementary table II. Time spent on different activity (min/d and steps/d) over time in all participants, males and females.** | | | | | | | | | | | | |
| --- | --- | --- | --- | --- | --- | --- | --- | --- | --- | --- | --- | --- |
|  | Baseline | | | Week4 | | | Week8 | | | Week12 | | |
|  | All | Men | Women | All | Men | Women | All | Men | Women | All | Men | Women |
| Average MET | 1.23±0.03 | 1.32±0.05 | 1.15±0.04 | 1.19±0.03 | 1.23±0.54 | 1.15±0.04 | 1.22±0.04 | 1.34±0.06 | 1.10±0.05 | 1.29±0.07 | 1.31±0.11 | 1.26±0.06 |
| Sed Time | 1612±413 | 1123±675 | 2100±477 | 1153±430 | 1155±675 | 1150±534 | 1150±487 | 1133±758 | 1167±616 | 1136±872 | 1171±1510 | 1100±871 |
| Light Act | 207±21 | 207±34 | 206±24 | 214±21 | 198±33 | 230±24 | 202±23 | 201±36 | 202±29 | 215±37 | 183±65 | 247±37 |
| Mod Act | 79±11 | 98±18 | 59±13 | 56±11 | 60±18 | 52±14 | 75±12 | 103±19 | 47±15 | 84±20 | 78±35 | 90±20 |
| Vig Act | 1.7±0.6 | 3.3±1.0 | 0.1±0.7 | 0.7±0.6 | 1.3±1.0 | 0.2±0.8 | 2.0±0.7 | 3.4±1.1 | 0.7±0.9 | 0.2±1.2 | -2.3±2.1 | 1.9±1.2 |
| Total PA | 287±26 | 309±42 | 266±31 | 271±27 | 259±42 | 283±33 | 278±29 | 307±45 | 250±36 | 299±48 | 258±84 | 339±47 |
| Steps/day | 7727±1016 | 7811±1632 | 7642±1210 | 6630±1027 | 5583±1620 | 7678±1264 | 7527±1091 | 8393±1715 | 6661±1350 | 8537±1637 | 7887±1715 | 9187±1637 |
| Results are expressed as estimated marginal means±SEM. MET: metabolic equivalent. Sed: sedentary. PA: physical activity. No significant main effect of time or time*sex interaction were found. Main effect of sex showed that males had higher MET (1.3±0.04 min/d vs. 1.16±0.03min/d, P<0.05) and spent more time on vigorous activity (2.36±0.64 min/d vs. 0.53±0.49 min/d (P<0.05). | | | | | | | | | | | | |
